# Systematic review of reviews on Activities of Daily Living measures for children with developmental disabilities

**DOI:** 10.1016/j.heliyon.2022.e09698

**Published:** 2022-06-13

**Authors:** Mo Chen, Anuradha S. Dutt, Rahul Nair

**Affiliations:** a Office of Education Research, National Institute of Education, Nanyang Technological University, 1 Nanyang Walk, 637616, Singapore; b Psychology, and Child and Human Development, National Institute of Education, Nanyang Technological University, 1 Nanyang Walk, 637616, Singapore; c Department of Dentistry—Quality and Safety of Oral Healthcare, Radboud University Medical Center, Radboud Institute for Health Sciences, Nijmegen, the Netherlands

**Keywords:** Developmental disability, Inclusive practices, Life skills

## Abstract

**Background:**

There seems to be a lack of consensus on the concept and domains of Activities of Daily Living (ADL) measured among children and adolescents with developmental disabilities (DD), despite a significant number of existing measures of ADL and associated constructs, and two prevailing theoretical frameworks (i.e., the cognitive-social-practical framework, and the activity-and-participation framework).

**Aims:**

This systematic review (SR) aims to identify articles that systematically reviewed measures of ADL for children and adolescents aged 7–18 years with DD to evaluate the quality of included articles, and describe the measures and domains identified.

**Methods:**

and Procedures: Searches were conducted in PubMed®, Academic Search Complete® (EBSCOhost), Education Source Search® (EBSCOhost), ERIC® (EBSCOhost), and PsychInfo® (EBSCOhost). 14,931 articles were identified, and two researchers completed title screening, abstract screening, and full-text screening, with disagreements resolved. Out of these 14,931 articles, fourteen were included, which resulted in a total of 163 ADL measures. Out of the 163 ADL measures, forty-eight met the criteria and were included for analysis. PRISMA and COSMIN checklists were used to appraise the methodological quality of the included articles.

**Outcomes and results:**

Results indicated that most of the 14 systematic review articles did not provide information on instrument development and content validity of their included measures. Analysis of the identified 48 measures of ADL and its associated constructs revealed heterogeneity in domains covered, although there were seven domains that were most often included.

**Conclusions and implications:**

Implications in terms of practice, research, and policy are further discussed.

**What this paper adds?**

This paper assessed articles that reviewed Activities of Daily Living (ADL) measures applied to children and adolescents with developmental disabilities aged 7–18 years old. Fourteen included studies were appraised for reporting and methodological quality according to recommendations in PRISMA and COSMIN. Key information (e.g., purpose, domains, outcome[s], and response format[s]) were extracted for the identified 48 measures of ADL and its associated constructs. Results suggest a lack of guidelines for quantitatively synthesizing SRs of measures and evaluating their psychometric properties. Using the COSMIN checklist, it was found that most reviews did not provide information on instrument development and details of content validity. Among the identified measures, each had seven domains on average. The key domains are personal/domestic, motor/mobility, academics, social, leisure, community participation, and communication. The most frequently measured outcomes were degree of competence in skills, degree of assistance required, frequency of behavior/activity performance, and degree of difficulty doing a task/activity. Rating scales were the most widely used response format. An evidence-based approach as delineated in this study may be conducive to understanding how ADL has been empirically represented in measurement practices.

## 1 Introduction

Activities of Daily Living (ADL) as a term, was first coined by Sidney Katz in 1950 in his field of gerontology and health services (Katz, 1983; Katz et al., 1970). It refers to a collection of activities in one's life spanning from basic ADL, such as eating, clothing, bathing, and mobility, to instrumental ADL, such as food preparation, shopping, taking medication, and transportation, and advanced ADL, such as leisure and working (Takechi et al., 2012). Since ADL serves as an indicator of a person's functional status, it is often related to some other terms or constructs, such as functional skills, and functional impairment.

ADL is also closely related to the construct of adaptive behavior or adaptive functioning. The similarity or resemblance can be reflected in one frequently cited definition for adaptive behavior or adaptive functioning - “the level of everyday performance of tasks that is required for a person to fulfill typical roles in society, including maintaining independence and meeting cultural expectations regarding personal and social responsibility” (VandenBos, 2006, p. 18). Notably, the construct of adaptive behavior evolved in the field of psychology in the 1950s when the notion was advocated that the classification of intellectual disability should not be solely relied on the assessment of intelligence quotient (IQ) (Saulnier and Klaiman, 2018).

Despite stemming from relatively different disciplinary foci (i.e., rehabilitation and health services vs. psychology), similar to measures of adaptive behavior, ADL measures aim to assess an individual's level of independence or perceived autonomy in performing different basic or more advanced activities necessary for daily living such as personal hygiene, socialization, communication, and community participation (James et al., 2014; Van der Linde et al., 2015). The key difference may be that ADL measures tend to be involved in a wider range of fields other than the field of psychological assessment (as the case for measures of adaptive behavior). Hence, the measurement of ADL is one of the most essential and practical measurements in disability assessment that spans across various fields of health, pediatrics, education, psychology, and rehabilitation (Field et al., 2016; Floyd et al., 2015; Jekel et al., 2015; Phillips et al., 2013).

### 1.1 Importance of ADL measures for populations with a disability

Using ADL measures for populations experiencing disability has considerable implications. From a broader perspective, ADL measures could be used to collect aggregate data on disability populations to inform international policies for a more inclusive society, as envisioned in the United Nations 2030 Agenda for Sustainable Development Goals (United Nations, 2015). The agenda aims towards building a globally inclusive society whereby equal access, opportunities, and infrastructural modifications are available to all individuals (including individuals with disability) in sectors of health, employment, education, gender equality, citizenship, etc.

Considering a narrower perspective, measures of adaptive behavior in the field of psychological assessment are used for diagnostic purposes to inform high-stakes decisions on needs and supports for individuals with disability at different developmental stages, such as informing a determination on whether a person can be diagnosed as having intellectual disability (American Psychological Association [APA], 2013). Such critical decisions also include informing educational placement alternatives and interventions, and accommodations to improve levels of self-sufficiency in different facets of daily living (APA, 2013; Schalock, 2012; Tassé et al., 2012).

It is noteworthy that in the field of psychology, measures of adaptive behavior have often been developed under one of two major theoretical frameworks. For one, the American Association on Intellectual and Developmental Disabilities (AAIDD) defines adaptive behavior in terms of three areas – conceptual, which includes language, numeracy, self-direction etc., social, which involves aspects of social problem solving, interpersonal skills, social responsibility etc., and practical skills which includes domains of personal care, vocational skills, community participation, healthcare etc. (cf. Tassé et al., 2012). Alternatively, according to the International Classification of Functioning (ICF), adaptive behavior is defined as activities and participation, which includes domains such as learning and applying knowledge, general tasks and demands, communication, mobility, self-care, domestic life, interpersonal interactions and relationships, major life areas, and community, social and civic life (World Health Organization [WHO], 2007).

On the basis of these two top-to-down theoretical frameworks, by far, more than 200 measures of adaptive behavior have been developed for different purposes (Saulnier and Klaiman, 2018). Meanwhile, efforts on developing new measures of adaptive behavior are underway globally, especially given increasing awareness of cultural relevance in ADL and advances in modern measurement theory (e.g., Chen et al., 2022).

Compared to measures of adaptive behavior in the field of psychology with a theoretically driven approach to measure development, there seems to be a lack of theoretical frameworks for measures of ADL, which are used across more various disciplinary fields. Hence, there remains a gray area where it is universally unclear about what domains constitute ADL (Kaur et al., 2016), within the broader perspective towards the well-being of all individuals across various fields, diverse cultures, and countries.

### 1.2 Purpose of the current study

As an initial step to understand the ADL measures especially for individuals with developmental disabilities (DD), the current study aims to take a down-to-top approach by first identifying ADL measures for children and adolescents with DD via a systematic approach and then analyzing the key attributes of these ADL measures (e.g., the domains covered). In particular, we decided to conduct a systematic review (SR) of articles that reviewed ADL measures. This decision was made on basis of two major rationales, (a) to identify the most relevant measures because the measures identified in the existing reviews of ADL measures have been scientifically endorsed to a certain degree in terms of psychometric evidence or practical utility, and (b) to avoid duplicates of existing literature reviews.

In accordance with the Centers for Disease Control and Prevention (CDC, n.d.), DD encompasses a group of conditions such as autism spectrum disorder (ASD), Attention-Deficit/Hyperactivity Disorder (ADHD), cerebral palsy, hearing loss, intellectual disability, learning disability, vision impairment, and other developmental delays. These conditions begin during the developmental period, arise from an impairment in physical, learning, language, or behavioral areas, and usually last throughout one's lifetime. The population of children and adolescents aged 7–18 years with DD was selected as significant placement, accommodation, and intervention support decisions are made based on ADL measures at these school aged years. Such decisions have a bearing on their current and future levels of functioning and development as they gradually transition into adulthood. Additionally, the school aged years also provide a wider range of daily activities across the home, school, and community settings that require to be learned and performed by individuals with DD to support participation in their social environment.

This study aims to (a) describe and assess the quality of reporting and evaluation of measurement properties in existing SRs identified, and (b) identify existing measures pertaining to ADL or its associated constructs for children and adolescents with DD aged 7–18 years to report various domains and outcomes measured. To the best of our knowledge, such an extensive review of measures in ADL or its associated constructs has not been conducted to inform the extent of psychometrically validated measures across multiple specialties in healthcare for school aged children and adolescents with DD.

## 2 Method

### 2.1 Inclusion and exclusion criteria

The included SRs had at least one study that described activities required for daily functioning based on self or others' reports (e.g., parents, teachers, therapists, etc.) for children and adolescents with DD aged 7–18 years. Table 1 shows more details on the inclusion and exclusion criteria used in this study to categorize the search results. Here, the first set of inclusion and exclusion criteria was used to decide whether a systematic review article should be included or excluded. Subsequently, for all the measures extracted from the included systematic review articles, we applied the second set of inclusion and exclusion criteria to decide whether a measure should be included or excluded for review.

**Table 1:** Inclusion and exclusion criteria for articles and measures.

**Inclusion criteria:**
• was a systematic review that described the systematic search procedures • reviewed measurement instruments that directly assess domains/aspects of daily living skills • reviewed the psychometric properties (commonly measures of reliability and validity) of the identified measurement instruments • target population of the reviewed instruments for individuals with developmental disabilities or special educational needs aged 7–18 years old. The articles were included as long as they included any part of this age group. • published in peer-reviewed journals • written or translated in English or Chinese as the authors were proficient in one or both languages.
**An article was excluded if it met one or more of the following exclusion criteria:**
• was not a systematic review that described the systematic search procedures • did not review measurement instruments that directly assess domains/aspects of daily living skills o only reviewed instruments that only assessed one domain/aspect of daily living skills (e.g., self-care) o only reviewed instruments that assessed a narrow aspect that was indirectly associated with daily living skills (e.g., motor skills, arm activity, hand use, upper limbs, gait, frailty) or a broad aspect that was indirectly associated with daily living skills (e.g., quality of life) • did not review the psychometric properties of the measurement instruments identified • the target population of the reviewed instruments for individuals with developmental disabilities or special educational needs aged 7–18 years old. Articles were excluded if they did not cover any part of the age range. o focused on chronic medical conditions (e.g., dementia, arthritis, diabetes, cancer, Parkinson's disease, pain, multiple sclerosis, stroke) o focused on mental health (e.g., mental illness, drug abuse/addiction) o focused on other non-developmental disabilities (e.g., aphasia, spinal cord injury) o focused on adults o focused on children younger than 7 years of age • commentaries, dissertations, protocols, book chapters
**For a measure to be included as references for ADL or its associated constructs, it should be:**
• reported in the identified systematic reviews via the current study • measured at least one aspect of daily living skills • had both reliability and validity evidence on those items related to daily living skills • applied to individuals with developmental disabilities aged 7–18 years old. Measures were included if they covered any part of the age range. • for some instruments (e.g., Vineland Adaptive Behavior Scale), multiple versions were developed over time. In this scenario, only the latest version of the instrument would be included for further analysis. If earlier versions were shown in the identified systematic reviews, it was replaced by the latest version. Also, for measures having two versions based on respondents (e.g., parent version vs. teacher version), both versions were included. • for instruments that were culturally validated, only the original one was included.
**For a measure to be excluded as references for ADL or its associated constructs, if it is:**
• a replication • targeted solely at individuals below 7 years old or at adults • measures other remotely relevant constructs, e.g., quality of life • measures only one specific area that is not directly targeted at daily living skills (e.g., motor skills [Bruininks-Oseretsky Test of Motor Performance –2]; hand use [Children's Hand-use Experience Questionnaire]; health/illness status [Child Health and Illness Profile – Child Edition]) • a generic tool, not directly related to daily living skills (e.g., Goal Attainment Scaling)

### 2.2 Search procedures

This SR of SRs complies with Preferred Reporting Items for Systematic Review and Meta-Analysis (PRISMA) guidelines (Moher et al., 2009). PRISMA is the recognized standard for reporting evidence in systematic reviews and meta-analyses. It consists of a 27-item checklist and a 4-phase flow diagram. The reviewer protocol for this article was registered in the International Prospective Register of Systematic Reviews (PROSPERO; CRD42020181365). Following initial searches for keywords, a comprehensive search strategy for five databases (a) Academic Search Complete via EBSCOhost, (b) Education Resources Information Center (ERIC; Access via EBSCOhost), (c) Education Source via EBSCOhost, (d) PsycINFO, and (e) PubMed, was performed from database inception to July 2020. There were no specifications for the date of publication. Key search terms were separated into four groups to represent four distinctive major concepts (i.e., adaptive behavior skills, scale, disabilities, and review). Alternative keywords for each central concept were included and linked in the search statement with Boolean operator OR. The four main concepts were linked via the Boolean operator AND to ensure the coverage of relevant articles. The online bibliographic tool Rayyan (Ouzzani et al., 2016) was used to manage all identified materials.

### 2.3 Selection of articles

**Figure 1:**
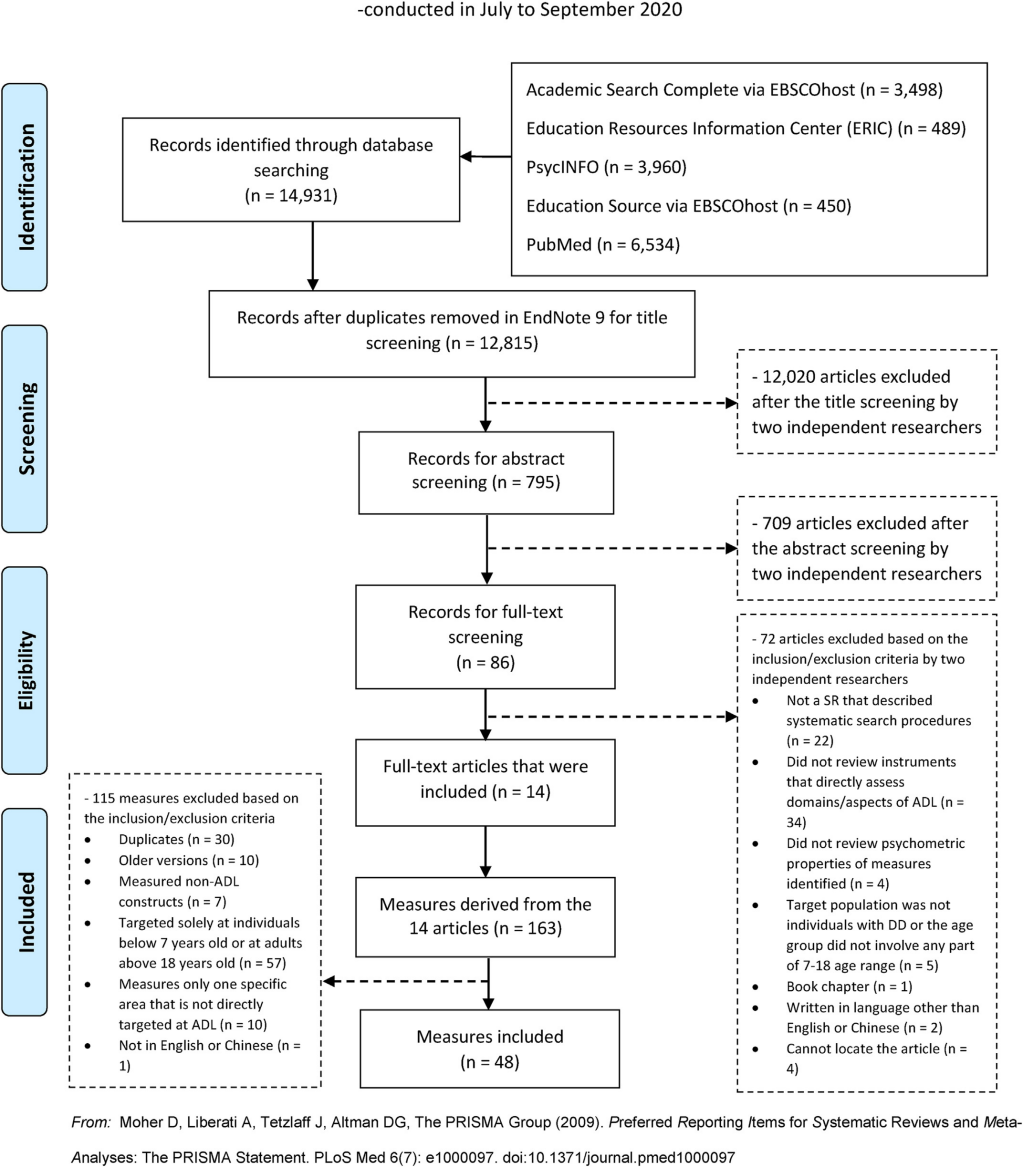
PRISMA flow diagram.

The searches were completed in July 2020, with 14,931 articles identified. With the duplicates removed in EndNote 9, there was a total of 12,815 articles for screening. Two researchers independently screened the title and abstract, resulting in 798 articles, with a Cohen's Kappa of 0.94. All disagreements were resolved via discussion, and 86 articles were eligible for full-text screening. We could not locate the full text for four of the 86 articles despite electronic searches and interlibrary loans through three international universities. Two researchers independently conducted full-text screenings of the 82 articles, with all disagreements resolved via discussion. Eventually, 14 articles met the inclusion criteria. Measures from these 14 SRs were extracted, resulting in a total of 163 entries. Following the inclusion and exclusion criteria, 48 measures were included for analysis (Table 1). The PRISMA flowchart is presented in Figure 1.

### 2.4 Quality assessment of the included SRs

Quality assessments for SRs such as the Assessment of Multiple Systematic Reviews (AMSTAR 2; Shea et al., 2017) are restricted to reviews pertaining to intervention, while methodological quality assessment of psychometric properties of individual measures within an SR could be conducted using the Consensus-based Standards for the selection of health Measurement Instruments (COSMIN; Prinsen et al., 2018). As these checklists did not directly apply to this SR of SRs that synthesized the psychometric properties of measures pertaining to ADL and its associated constructs, this study assessed the extent of application of the measurement properties outlined in the COSMIN consensus report for the included SRs. Additionally, the PRISMA checklist was used to assess the extent of reporting covered within the SRs. In the PRISMA checklist, questions explicitly addressed details on participants, interventions, comparisons, outcomes, and the study design. Since our included studies were SRs of assessment measures, we gauged if an article described its objectives based on an assessment of its psychometric properties. Similarly, the criteria for summary measures and synthesis of measures in PRISMA accounted for measures of validity, reliability, and responsiveness.

### 2.5 Data extraction

Data extraction forms were created with consensus among the research team, and two researchers carried out data extraction independently. Any differences that were found were then discussed with a third researcher and resolved. PRISMA and COSMIN checklists were used to code each of the 14 articles by two researchers independently. Two researchers checked and discussed each disagreement until a consensus was reached. Basic information of the identified measures was extracted and coded by a researcher descriptively and subsequently crossed checked by another independent researcher.

## 3 Results

### 3.1 Description of included 14 SR articles

As Table 2 shows, the target constructs of the measures synthesized across the 14 articles were focused on ADL and its associated constructs such as adaptive behavior, adaptive functioning activities and participation, functional skills/impairments, etc. Across the 14 articles, the number of included studies ranged from seven to 138, with four articles not indicating the number of studies. The number of measures included on ADL, or its associated constructs ranged from four to 31. Five articles focused on measures for children/individuals with cerebral palsy. Five other articles included measures for children/individuals with disabilities not focusing on a particular type or diagnosis. Other articles (i.e., one article for each disability population) contained measures for children/individuals with power mobility needs, developmental coordination disorder, Rett Syndrome, and mild cognitive impairments. The seven articles focusing on a specific diagnosis (i.e., cerebral palsy, developmental coordination disorder, and Rett syndrome) reported four to eight measures. The other seven articles not targeting a specific disability group reported nine to 31 measures.

**Table 2:** Description of included review articles.

Citations	Target Construct (Population)	Information Sources	# of Included Studies	# of Included Instruments
Ferre-Fernández et al. (2020)	Motor and functional skills (for children with cerebral palsy)	Pubmed/MEDLINE; ISI Web of Science; Science Direct; CINAHL; PEDro; Biblioteca Virtual de la Salud (BVS)	12	4
Semmel et al. (2019)	Adaptive functioning (for individuals with Rett Syndrome)	Pub-Med	23	7
Field et al. (2016)	Participation (for children and youth with power mobility needs)	Identified primary peer-reviewed studies and systematic reviews published from databases: CINAHL; EBM Reviews; EMBASE; ERIC; Health and Psychosocial Instruments; MEDLINE (Ovid SP); OTseeker; PEDro; PsycINFO; electronic tool and author searches, and hand search of reference lists of identified articles	138	20
Floyd et al. (2015)	Adaptive behavior (for children and adolescents who experience a range of disabling conditions)	Reference texts, book chapters, testing information clearinghouses, and assessment catalogs	Not mentioned	14
Jekel et al. (2015)	Deficits in Instrumental activities of daily living (for patients with mild cognitive impairment)	PubMed; Web of Science; PsycINFO	37	31
van der Linde et al. (2015)	Activities of daily living (for children with developmental coordination disorder)	MEDLINE; EMBASE; CINAHL; PsycINFO	66	7
James et al. (2014)	Activities of daily living (for children and adolescents with cerebral palsy)	Pub-Med; EMBASE; CINAHL; PsycINFO; Cochrane Library; hand search of reference lists of key papers	49	8
Phillips et al. (2013)	Activity and participation (for children and adolescents with disabilities)	AMED; MEDLINE; EMBASE; CINAHL; Scopus; Web of Knowledge; PubMed	116	20
Noonan et al. (2009)	Participation (for individuals with various health conditions)	Medline; CINAHL; EMBASE; HaPI; Psyc (Info, Articles, Books); reference lists of all research articles selected	52	11
Harvey et al. (2008)	Activity limitation (for children with cerebral palsy)	MEDLINE; EMBASE; CINAHL; PsycINFO; hand search of reference lists from key articles and key journals	29	8
Palermo et al., 2008a, Palermo et al., 2008b	Health-related quality of life and functional impairment (for children and adolescents in general, with various conditions or functional impairment)	review of recent textbooks and review articles on assessment of HRQOL by workgroups of Society of Pediatric Psychology Assessment Task Force	Not mentioned	16
Sakzewski et al. (2007)	Participation (for children with cerebral palsy)	Medline; CINAHL; EMBASE; PsycINFO, assessment manuals	Not mentioned	7
Morris et al. (2005)	Activity performance and participation (for children with cerebral palsy)	Medline; EMBASE; CINAHL; AMED; PsycINFO, Sociofile and the PAHOP database of Patient-assessed Health Instruments; reference lists of identified articles and reviews	7	7
Perenboom and Chorus (2003)	Participation (for individuals with handicap)	MEDLINE	Not mentioned	9

### 3.2 Reporting quality among the included SRs (PRISMA)

Twelve of 14 articles identified themselves as an SR or meta-analysis in the title (Table 3). All 14 articles provided a structured summary in the abstract. They also described the rationale in the review and provided an explicit statement on research questions or objectives within the introduction. Only one article (Ferre-Fernández et al., 2020) indicated details on its registration, review protocol, and where it could be accessed.

**Table 3:** PRISMA coding results for the 14 articles.

Citation	Title	Abstract	Introduction		Methods												Results							Discussion			Funding
			Rationale	Objectives	Protocol and registration	Eligibility criteria	Information sources	Search	Study selection	Data collection process	Data items	Risk of bias in individual studies	Summary measures	Synthesis of results	Risk of bias across studies	Additional analyses	Study selection	Study characteristics	Risk of bias within studies	Results of individual studies	Synthesis of results	Risk of bias across studies	Additional analysis	Summary of evidence	Limitations	Conclusions	
Ferre-Fernández et al. (2020)	+	+	+	+	+	+	+	+	+	+	-	+	-	-	*-*	+	+	+	+	+	-	*-*	+	+	+	+	-
Semmel et al. (2019)	+	+	+	+	-	+	-	-	+	-	+	+	-	-	*-*	-	+	+	+	+	-	*-*	-	+	+	+	+
Field et al. (2016)	+	+	+	+	-	+	+	+	+	+	-	+	-	-	*-*	-	+	+	+	+	-	*-*	-	+	+	+	-
Floyd et al. (2015)	+	+	+	+	-	+	+	-	+	+	+	+	-	-	*-*	-	-	+	+	+	-	*-*	-	+	+	+	-
Jekel et al. (2015)	+	+	+	+	-	+	+	+	+	-	-	-	-	-	*-*	-	+	+	-	+	-	*-*	-	+	-	+	+
van der Linde et al. (2015)	+	+	+	+	-	+	+	+	+	-	-	-	-	-	*-*	-	+	+	-	+	-	*-*	-	+	+	+	+
James et al. (2014)	+	+	+	+	-	+	+	+	+	+	+	+	-	-	*-*	-	+	+	+	+	-	*-*	-	+	+	+	+
Phillips et al. (2013)	+	+	+	+	-	-	+	+	+	-	-	+	-	-	*-*	-	-	+	+	+	-	*-*	-	+	+	+	-
Noonan et al. (2009)	+	+	+	+	-	+	+	+	-	-	+	+	-	-	*-*	-	-	+	+	+	-	*-*	-	+	+	+	+
Harvey et al. (2008)	+	+	+	+	-	+	+	+	+	+	-	+	-	-	*-*	-	+	+	+	+	-	*-*	-	+	-	+	+
Palermo et al., 2008a, Palermo et al., 2008b	-	+	+	+	-	+	-	-	-	+	+	+	-	-	*-*	-	-	+	+	+	-	*-*	-	+	+	+	-
Sakzewski et al. (2007)	+	+	+	+	-	+	+	+	+	+	+	+	-	-	*-*	-	+	+	+	+	-	*-*	-	+	+	+	+
Morris et al. (2005)	+	+	+	+	-	+	+	+	-	-	+	+	-	-	*-*	-	+	+	+	+	-	*-*	-	+	-	+	-
Perenboom and Chorus (2003)	-	+	+	+	-	-	-	+	-	-	-	-	-	-	*-*	-	-	-	-	-	-	*-*	-	+	-	-	-

Note: (+= Yes, - = No).

Twelve articles specified study characteristics and reported characteristics (e.g., years considered, language, publication status) used as eligibility criteria, with a rationale provided. Eleven articles described all information sources in the search, including the last search date. Eleven articles presented a complete electronic search strategy for at least one database. Ten articles stated the process for study selection. Seven studies described the data collection process (e.g., how the data were extracted). Seven articles listed and defined the variables for which data were coded. Eleven articles described methods used to assess individual studies' measurement properties and how this information could be used in data synthesis. None of the articles stated principal summary measures (e.g., summative measures reporting the reliability or validity evidence across the studies within a systematic review). Similarly, none of the articles described the methods of handling data and combining results of studies or specified any assessment of risk of bias that may affect cumulative evidence (e.g., publication bias, selective reporting within studies).

Within the results, nine articles provided details on the total numbers of studies screened, studies included in the review based on an eligibility assessment, and reasons for exclusions at each stage. Five articles presented a flow diagram delineating the inclusion and exclusion process of the studies (Ferre-Fernández et al., 2020; Field et al., 2016; James et al., 2014; Semmel et al., 2019; Van der Linde et al., 2015). Thirteen articles reported characteristics for which data were extracted, with citations provided. Eleven articles reported data on the risk of bias for each study. All 14 articles except one article (Perenboom and Chorus, 2003) reported the psychometric results of individual measures identified. None of the studies reported a synthesis of results or results pertaining to the assessment of risk of bias across studies.

For the discussion section, all 14 articles provided a summary of the main findings; 10 articles discussed limitations at individual study and review levels; 13 articles contained a conclusion giving a general interpretation of the results. Finally, seven articles described sources of funding for the SR.

### 3.3 COSMIN results: adequacy of measurement properties reported in the 14 SRs

Table 4 shows that none of the articles examined the instrument development of the included measures. Among the included articles, seven used self-developed checklists, while the rest (n = 7) used pre-developed criteria. Two publications (Ferre-Fernández et al., 2020; Field et al., 2016) used COSMIN criteria. Other articles used criteria such as the modified CanChild Outcome Measures Rating Form (James et al., 2014), the Medical Outcomes Trust (MOT; Noonan et al., 2009), and the Standards for Educational and Psychological Testing (Floyd et al., 2015). One article (Field et al., 2016) examined content validity while another article (Floyd et al., 2015) partially examined content validity. The remaining 12 articles did not report details regarding the content validity of the measures. Half of the articles examined the structural validity of the measures. All articles except two (Perenboom and Chorus, 2003; Semmel et al., 2019) examined the internal consistency and reliability of the measures. Three articles (Ferre-Fernández et al., 2020; Field et al., 2016; Noonan et al., 2009) examined cross-cultural validity or measurement invariance, as well as measurement error of the measures. Four articles reported criterion validity. Ten articles reported hypothesis testing for the construct validity of measures, and eight articles reported responsiveness of the included measures.

**Table 4:** COSMIN coding results: Adequacy of measurement properties.

Citations	Instrument Development	Content Validity	Structural Validity	Internal Consistency	Cross-Cultural Validity/Measurement Invariance	Reliability	Measurement Error	Criterion Validity	Hypothesis Testing for Construct Validity	Responsiveness
Ferre-Fernández et al. (2020)	−	−	+	+	+	+	+	−	+	+
Semmel et al. (2019)	−	−	−	−	−	−	−	−	−	−
Field et al. (2016)	−	+	+	+	+	+	+	+	+	+
Floyd et al. (2015)	−	±	+	+	−	+	−	+	−	−
Jekel et al. (2015)	−	−	−	+	−	+	−	−	+	−
van der Linde et al. (2015)	−	−	−	+	−	+	−	−	+	−
James et al. (2014)	−	−	+	+	−	+	−	+	−	+
Phillips et al. (2013)	−	−	−	+	−	+	−	−	+	−
Noonan et al. (2009)	−	−	+	+	+	+	+	−	+	+
Harvey et al. (2008)	−	−	?	+	−	+	−	−	+	+
Palermo et al., 2008a, Palermo et al., 2008b	−	−	+	+	−	+	−	−	+	+
Sakzewski et al. (2007)	−	−	+	+	−	+	−	+	+	+
Morris et al. (2005)	−	−	−	+	−	+	−	−	+	+
Perenboom and Chorus (2003)	−	−	−	−	−	−	−	−	−	−

Note: (+= Yes, − = No, ± = Maybe, ? = Uncertain).

### 3.4 Domains of the included measures across the 14 review articles

Across the 48 measures, the number of domains across 47 measures totaled 313, ranging from 1 to 24 (*M* = 6.7, *SD* = 4.0), with the domains for one measure (i.e., the *Quality of Social Functioning Scale and Index*) not explicitly stated. The number of items ranging from 8 to 502 (*M* = 87.3, *SD* = 109.8), and thus the number of items per domain varied widely across the 47 measures, ranging from 2 to 100 (*M* = 15.5, *SD* = 20.1). The 313 domains could be further categorized into 19 broader domains. The most frequently occurring domains involved personal/domestic, motor skills/mobility, academics, social, leisure, community participation, and communication.

Additionally, the purpose or intended use of these measures usually involved assessing levels of independence with performing a wide variety of activities for daily functioning or targeted assessment in more specific areas (e.g., motor skills, occupational performance, pain, etc.). Twenty nine out of the 48 measures are freely downloadable or could be requested from the developer(s), while the other 19 measures require to be commercially purchased. Finally, 19 of the 48 ADL measures assessed one outcome while the other 29 measures assessed more than one outcome (range: 2–34). The ADL outcomes assessed included degree of competence in skills, degree of difficulty in completing a task/activity, degree of assistance required, frequency of activity participation, degree of importance, degree of satisfaction, amount spent in activity, etc. The most frequently measured outcomes were degree of competence in skills, degree of assistance required, frequency of behavior/activity performance, and degree of difficulty doing a task/activity. In terms of response formats, rating scales, multiple choice items, and open-ended items were used across the 48 measures. Rating scales were the most widely used response format.

## 4 Discussion

This study conducted an SR of SRs that synthesized measures assessing ADL for children and adolescents with DD aged 7–18 years. Fourteen SRs were identified from the initial search output of 14,931 articles, and 48 measures were further identified among the 163 measures derived from the included studies. Aligned with research literature on ADL measures for children and adolescents with DD, SRs in the current paper reported consistent trends in assessing psychometric properties of the individual measures, with common areas of deficits and strengths (Floyd et al., 2015; James et al., 2014; Palermo et al., 2008a, Palermo et al., 2008b). These instruments reported on 314 domains (further categorized into 19 domains), which targeted areas of daily functioning or specific skills pertaining to daily living. An analysis of the domains covered across the 48 measures indicated an average of 6–7 domains for each measure and various ADL response outcomes (e.g., degree of competence in skills, degree of difficulty in completing a task/activity, degree of assistance required etc.). Our search suggests that the assessment of independence levels in ADL was commonly observed in areas of personal/domestic, motor skills/mobility, academics, social, leisure, community participation, and communication (Jekel et al., 2015; Noonan et al., 2009; Phillips et al., 2013; Semmel et al., 2019).

The 14 SRs of measurement instruments could not meet all criteria outlined in the PRISMA checklist. A consistent deficit with reporting results across the 14 SRs was the lack of information on summary measures, the synthesis of results (i.e., principal summary measures), and the overall risk of bias on the studies reviewed based on PRISMA standards. This could be due to an abundance of statistics in psychometrics, heterogeneity of instruments with different theoretical frameworks as reflected in their varied purposes, and the lack of well-understood methodologies for meta-analysis of psychometrics-related studies. However, when reviewers selectively choose which information to include in a review based on the direction and significance of findings, they risk biasing the evidence base on which healthcare decisions and policies are made. Hence, the next logical and imperative step for research in this area is to employ a well-established criterion, such as the PRISMA checklist, to critically evaluate studies and avoid biases in reporting outcomes within reviews (Shamseer et al., 2015).

The evaluation of measurement properties by the included systematic reviews often used self-developed checklists. Only two of the remaining seven publications used a common checklist (COSMIN), and the rest used various criteria for evaluating the measurement properties of their included ADL measures. This introduces considerable heterogeneity to their review methodology as well as inherent difficulties in comparing the findings of these studies. The evaluation of SRs using the COSMIN checklist showed that most review articles reported psychometric properties of studies included in terms of reliability, construct validity, and responsiveness but lacked methodological rigor (Kaur et al., 2016). For instance, most of the review articles lacked information on instrument development and details on content validity. This omission suggests the difficulty in assessing these areas and could also be a reason for variations in the domains that emerged from the included measures. This omission was also observed in instruments that followed the domains prescribed by the ICF, AAIDD, or Diagnostic Statistical Manual – 5^th^ edition (DSM-5). Providing details of instrument development would give us crucial information regarding the relevance, comprehensiveness, and comprehensibility of the measures (Terwee et al., 2018).

Based on our review, this article could be the first of its kind to provide a comprehensive overview of assessment measures pertaining to ADL and its associated constructs for children and adolescents with DD aged 7–18 years. The relevance of this systematic search is three-fold. Firstly, for practitioners working closely with children and adolescents with DD, this article provides an extensive inventory of measures that could be used to assess and monitor practical outcomes for this population. Secondly, this consolidated database could provide a foundation for researchers and scale developers to generate future ADL measures while incorporating newer items or domains across various aspects of daily functioning to keep abreast with universal technological advancements (e.g., items on digital literacy). Finally, for policymakers trying to reach global targets for a more inclusive world, this register of measures (including 29 freely available measures) could help improve accessibility to assessment options for ADLs across countries, families, and children and adolescents with DD where resources are limited.

It is noteworthy that the purpose of this paper was not to identify an ideal or perfect ADL measure. Employing a specific instrument may differ considering client contextual variables such as their primary needs (e.g., language, socio-economic status, gender, etc.), their context's cultural attitudes and beliefs towards disability, support based on national/global policies etc. (Scior, 2011). The aim of this paper was to provide a comprehensive repository of ADL measures for children and adolescents with DD (aged 7–18 years) that could be used by practitioners, researchers, and policy makers to guide intervention support, accommodations, research, and policy for this population in light of global and national objectives for disability. Hence, caution should be taken to assess if an instrument serves all purposes of ADL measurement considering an individual's contextual relevance.

### 4.1 Limitations and future research directions

Although we appraised the included SRs, we did not systematically evaluate the psychometric properties of each measurement tool since it was not the focus of this investigation. Future research could conduct a more in-depth analysis of the identified 48 measures to examine their psychometrics and other relevant features (e.g., types of rating scale used, the country where the measure was developed, etc.). Additionally, as this paper involved an SR of review articles, newer measures assessing ADL that are freely available or commercially purchased were not included. For example, AAIDD's *Diagnostic Adaptive Behavior Scale* (DABS; Tassé, 2017) was recently developed as a standardized assessment of adaptive behavior for individuals from 4 to 21 years old. It covers some skills that are rarely mentioned in the existing measures, such as gullibility, naïveté (i.e., wariness), digital literacy, etc. These newer measures could be captured in future systematic reviews pertaining to assessing ADL skills.

## 5 Conclusions

The findings of this SR of SRs exemplify a systematic approach to identifying measures focusing on ADL, with the aim of providing clarity to its operational definition. Focusing on the population of children and adolescents with DD aged 7–18 years old, the identified measures that primarily rely on others' reports examined ADL from different domains with a consistent purpose of gathering information to inform an individual's functional independence in day-to-day life. Without systematically documenting ADL performance for children and adolescents with DD using appropriate measures, it would be difficult to move forward toward the prospect of a more inclusive society, as outlined in the Sustainable Development Goals by the United Nations.

## Declarations

### Author contribution statement

Rahul Nair; Mo Chen; Anuradha SK Dutt: Conceived and designed the experiments; Analyzed and interpreted the data; Contributed reagents, materials, analysis tools or data; Wrote the paper.

### Funding statement

Mo Chen was supported by Office of Educational research- 10.13039/501100006124National Institute of Education, Singapore [DEV 05/19 CM].

### Data availability statement

The authors do not have permission to share data.

### Declaration of interest's statement

The authors declare no conflict of interest.

### Additional information

No additional information is available for this paper.
